# Identifying gaps in the care and management of NTD morbidity, disability and disfigurement in Africa

**DOI:** 10.1371/journal.pntd.0013834

**Published:** 2025-12-11

**Authors:** Lorraine Tsitsi Pfavayi, Derick Nii Mensah Osakunor, Crecencia Edward Chiombola, Francisca Mutapi

**Affiliations:** 1 Institute of Immunology and Infection Research, The University of Edinburgh, Edinburgh, United Kingdom; 2 Tackling Infections to Benefit Africa (TIBA) Partnership, The University of Edinburgh, Edinburgh, United Kingdom; 3 School of Biodiversity, One Health, and Veterinary Medicine, University of Glasgow, Glasgow, United Kingdom; 4 Department of Medical Parasitology and Entomology, School of Medicine, Catholic University of Health and Allied Sciences, Mwanza, Tanzania; 5 Tackling Infections to Benefit Africa (TIBA) Partnership, Catholic University of Health and Allied Sciences, Mwanza, Tanzania; IRCCS Sacro Cuore Don Calabria Hospital, ITALY

## Abstract

**Background:**

Neglected tropical diseases (NTDs) affect about 1.7 billion people worldwide, with the highest burden in sub-Saharan Africa. While global initiatives have reduced infection prevalence, the chronic impacts of NTDs, including morbidity, disability, and disfigurement, remain severely neglected, perpetuating poverty, social exclusion, and mental health challenges. This study aimed to identify gaps in care and management for individuals living with chronic NTD-related disabilities and to validate the situational analysis findings through participatory workshops.

**Methodology/Principal Findings:**

A mixed-methods approach was employed, comprising a situational analysis of national policies, healthcare services, and control strategies across the World Health Organization (WHO) African Region, complemented by participatory workshops, including focus group discussions (FGDs) and in-depth interviews (IDIs) with affected individuals and stakeholders, in Zimbabwe and Tanzania. Qualitative data were thematically analysed to capture key challenges, including healthcare access barriers, stigma, and gaps in care and management. The situational analysis revealed critical gaps in chronic NTD care, including lack of comprehensive policies for NTD disability management, fragmented health services, limited access to rehabilitation and mental health support, and where available at all, weak integration of morbidity management into national health systems. Findings from FGDs and IDIs corroborated these findings, highlighting barriers to healthcare access, policy deficiencies, socioeconomic burden, deep-rooted stigma, and psychological distress. Participants emphasised the urgency of integrating chronic care into national health systems, strengthening service delivery, and ensuring financial protection.

**Conclusions/Significance:**

Current NTD programs prioritise infection control and disease elimination while neglecting long-term disability management. To ensure end-to-end health service provision, it is imperative to integrate morbidity management, disability support, psychosocial support, and rehabilitation into existing healthcare frameworks, informed directly by the voices of those affected. Ultimately, by bridging the gaps between policy, healthcare, and community engagement, and mainstreaming NTD morbidity management, we can ensure that individuals affected by NTDs receive comprehensive, long-term support, and that progress in disease control translates into sustained improvements in well-being.

## Author summary

Neglected tropical diseases (NTDs) affect about 1.7 billion people globally, with the highest burden in sub-Saharan Africa. While efforts to control infections have achieved important milestones, less attention has been given to the long-term effects of NTDs, such as disability, disfigurement, and mental health conditions, which can persist long after treatment. These long-term consequences often go unrecognised in national health systems, leaving affected individuals to face stigma, poverty, and limited access to care. We reviewed national health policies in the World Health Organisation (WHO) African Region and conducted interviews and focus group discussions with people living with NTD-related chronic morbidity, healthcare workers, and other key stakeholders in Tanzania and Zimbabwe. We identified major gaps in care, including limited access to rehabilitation and mental health services, and poor integration of disability care into routine health services. Participants described facing social exclusion, stigma, and significant financial burdens that compounded the burden of living with diasbility from NTDs. Our study calls for a shift in NTD programmes to go beyond infection control and include long-term support and care. Strengthening health systems to provide rehabilitation, mental health care, and social protection, alongside engaging affected communities to shape inclusive policies and services that address their long-term needs, is essential to improving the long-term well-being.

## Introduction

Neglected tropical diseases (NTDs) are a diverse group of 21 disabling, avoidable, and debilitating conditions that continue to affect about 1.7 billion people worldwide [1,2], primarily in impoverished communities in tropical and subtropical regions [3]. Despite their high prevalence, NTDs are classified as “neglected” because they disproportionately impact the poor, and the regions affected have historically received limited attention and resources compared to other global health priorities [4,5].

NTDs are prevalent in vulnerable and marginalised communities where limited access to essential resources like safe water, sanitation, and healthcare exacerbate the burden [3,5]. While global efforts in prevention and control have led to a decline in the number of individuals affected, the chronic manifestations of NTDs, including morbidity, disability, and disfigurement, continue to affect millions, particularly in sub-Saharan Africa [6]. For many, the lifelong effects are not only physically debilitating but also socially isolating, compromising mental well-being, economic stability, and social integration [7].

Chronic NTDs account for an estimated 19 million disability-adjusted life years (DALYs) lost annually [3]. However, this figure likely underestimates their true impact, as it does not fully consider long-term chronic morbidities, the stigma, social exclusion, and the psychological toll that accompany these diseases [8–10]. For instance, in the case of lymphatic filariasis (LF), studies show that when depression is included, the total DALY burden nearly doubles, indicating that previous estimates have significantly underestimated the true impact of the disease [11]. Similarly, for cutaneous leishmaniasis, the burden is substantially higher when major depressive disorder is taken into account [12]. Moreover, DALYs primarily do not account for patient perspectives, chronic complications, as well as shared disabilities in the presence of comorbidities or concurrent infections in NTD-endemic areas [8]; thus, their actual impact on health and quality of life may be significantly higher than often reported.

**Fig 1:**
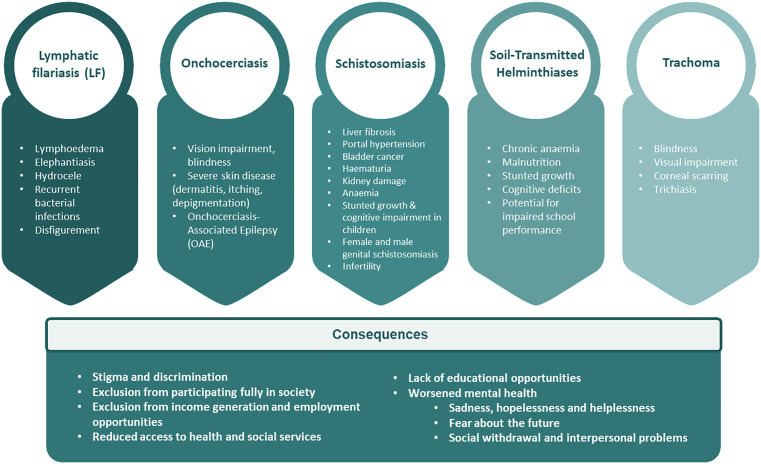
Preventive chemotherapy-targeted NTDs and their associated chronic morbidities. This Figure shows the chronic morbidities associated with Lymphatic filariasis (LF) [14], Onchocerciasis [15], schistosomiasis [16–20], soil-transmitted helminthiases [21] and trachoma [22] as well as their long-term consequences [23]. This figure was produced in PowerPoint.

The World Health Organization (WHO) has emphasised in the 2024 Global report on NTDs that “*It is not yet possible to assess the value of this indicator after the launch of the road map as the latest available data refer to 2019*” [13], highlighting that multiple limitations affect the tracking of DALYs for NTDs [10]. Some of these limitations include the lack of DALY estimates for some NTDs (e.g., Buruli ulcer, chikungunya, mycetoma) and for others (e.g., dracunculiasis and scabies), a lack of inclusion in the Global Health Estimates (GHE). Moreover, there is need to update methodologies and ensure that DALY estimates by GHE coincide with the latest WHO NTD Road map’s reporting requirements of every 4–5 years [10,13]. Many NTDs, particularly those with visible symptoms, such as LF, onchocerciasis, leprosy, Buruli ulcer, schistosomiasis, and trachoma, cause lifelong disability, disfigurement and pain, exacerbating social and economic hardships [5]. To illustrate this, Fig 1 summarises the five Preventive chemotherapy-targeted NTDs (PC-NTDs), and their associated chronic morbidities, emphasising the long-term health and socioeconomic burdens they impose.

While Fig 1 is not exhaustive of all NTDs included in this study, it demonstrates how chronic manifestations contribute to long-term health and socioeconomic burdens. For example, 1.2–5.5 million cases of snakebite envenoming have been reported annually, with more than 125,000 deaths and three to four times as many cases resulting in permanent disability or disfigurement [24–27]. In sub-Saharan Africa alone, annual incidence is estimated at 435,000–580,000 cases [28–30]; a regional analysis across 41 African countries reported approximately 1.03 million DALYs associated with snakebite, including 268,471 cases, 12,290 deaths, 14,766 amputations and around 55,332 cases of post-traumatic stress disorder [29,31]. However, the true burden, is likely higher as many incidents occur in rural areas where people affected often first seek treatment from traditional healers rather than formal health facilities, leading to poor documentation and substantial under-reporting in national health systems [29,32–34]. Given this significant but underrepresented burden, and the high snakebite envenoming prevalence in the study areas, there is a need to capture context-specific information to inform integrated NTD management approaches [35,36].

### Gaps in NTD care and management

Despite global progress in NTD control, significant challenges remain in managing the chronic sequelae of this group of diseases. Most affected individuals live in remote or rural areas with limited healthcare infrastructure, complicating access to diagnosis, treatment, and rehabilitation services. Additionally, local and national healthcare systems, often strained by other public health demands, are frequently ill-equipped to address the complex, long-term care needs of NTD patients [37–39].

While PC through mass drug administration (MDA) has successfully reduced transmission rates [40], it does not fully address the chronic pathology, disability, stigma, and mental health challenges faced by individuals with chronic NTD-related conditions [41]. It is thus not surprising that as of 2019, the latest NTD-related DALYs data show a global reduction of only 11% between 2015 and 2019, far from the 75% target set to be achieved by 2030 [10,42]. Additionally, issues of equity, autonomy and sustainability have been highlighted as domains with the most pressing ethical concerns in relation to the MDA for NTDs [43]. To help bridge this gap, initiatives such as the WHO’s Minimum Package of Care (MPC) and Morbidity Management and Disability Prevention (MMDP) programs have aimed to improve long-term patient outcomes by providing essential care for conditions such as lymphedema, elephantiasis, and hydrocele [44]. However, the extent of integration of these programs into national health systems remains inconsistent across many African countries; WHO provides toolkits and conducts workshops to support national programs in implementing MMDP services [45,46].

Furthermore, some PC programs to target specific at-risk groups (e.g., school-aged children) while others target the whole community [47,48].While this approach has reduced infection prevalence and morbidity in target populations, other at-risk adults, preschool-aged children, and marginalised groups often remain untreated, creating reservoirs of infection that sustain transmission [49]. These untreated populations not only perpetuate endemicity but also endure chronic infections, leading to severe, long-term pathologies that undermine broader disease elimination goals.

At the community level, support structures are often inadequate or non-existent, leading to further isolation of individuals living with NTD-related disabilities [43,50]. This isolation contributes to poor mental health outcomes, as affected individuals struggle with social stigma, loss of economic productivity, and limited opportunities for education or employment [51,52]. The cumulative effect of NTD-related morbidity, disability, and disfigurement perpetuates cycles of poverty and inequality, limiting the ability of affected individuals to contribute to their communities [53].

### The need for a situational analysis

Current NTD control frameworks must move beyond a vertical approach of infection control and morbidity reduction to a holistic and inclusive approach that addresses multiple chronic infections and their associated disabilities. Strengthening health systems to integrate NTD services across the entire patient journey, promoting sustainable surveillance mechanisms, and improving community engagement are crucial to achieving equitable healthcare access. Without such shifts, the burden of chronic NTD-related complications will continue to disproportionately affect neglected populations, reinforcing cycles of poverty and health disparity.

Therefore, understanding the gaps in NTD care and management in endemic areas from both a regional health system perspective and the lived experiences of affected individuals and healthcare providers is crucial. We conducted, a comprehensive situational analysis of the current healthcare services, policies, and existing data in NTD-endemic countries, to identify gaps in the care and management of individuals with chronic NTDs. Through focus group discussions and interviews this research also captures the lived experiences of individuals affected by the debilitating impacts of NTDs, highlighting inequities in healthcare access, and informing strategies for improving long-term support and social inclusion for individuals affected by NTDs. This study aimed to 1) conduct a situational analysis of health service provision for chronic NTD sufferers and identify gaps in care and management across WHO AFRO countries b) Validate situational analysis findings through focus group discussions (FGD) and in-depth interviews (IDIs) in 2 African countries.

## Methodology

### Study design

This study adopted a qualitative, mixed-methods approach to assess health service provision for chronic NTDs and identify gaps in care, management, and policy. This included **1) Situational analysis**: a comprehensive review of healthcare services, policies, and existing data to identify gaps in the care and management of individuals with chronic NTDs in WHO AFRO countries**, and 2) Consultative workshops**: participatory workshops in Tanzania and Zimbabwe involving FGD, IDIs, and stakeholder presentations to validate the situational analysis findings and incorporate perspectives from NTD-affected individuals, healthcare providers, policymakers, and researchers/ academics. This mixed approach allowed for a comprehensive understanding of current healthcare provisions while integrating first-hand experiences of affected individuals and healthcare providers.

### Situational analysis

The situational analysis examined broader regional trends across the WHO AFRO region to map general trends and systemic gaps. Data sources included websites and repositories of the main NTD actors in the WHO African region such as the WHO Regional Office for Africa (WHO AFRO; https://www.afro.who.int), WHO AFRO’s Expanded Special Project for Elimination of Neglected Tropical Diseases (ESPEN; https://espen.afro.who.int/regions/who-african-region-afro), Uniting to Combat NTDs (https://unitingtocombatntds.org/en/), national NTD master plans (https://espen.afro.who.int/tools-resources/documents) as well as published peer-reviewed literature on chronic NTD-related morbidity, disability, and social impact.

Additional information including data on 1) health service provision, 2) control strategies for the five PC-targeted NTDs (Lymphatic filariasis (LF), Trachoma, Onchocerciasis, Schistosomiasis and Soil transmitted helminthiases (STH)), 3) chronic care and management for the five PC-NTDs 4) chronic care and management for other NTDs outside of the five PC-targeted ones, 5) gaps in chronic care and management, were also extracted from the literature and additional sources.

### Consultative workshops

The consultative workshops focused specifically on Tanzania and Zimbabwe and prioritised the four most prevalent NTDs in both countries that also cause the highest prevalence of disability or disfigurement [54–56]. The workshops included FGDs, IDIs and stakeholder presentations to validate and expand on the findings from the situational analysis.

#### Ethical approval.

Ethical and institutional approval was obtained from the Medical Research Council of Zimbabwe (MRCZ/A/3030) and the National Ethical Review Committee board of Tanzania (NIMR/HQ/R.8a/Vol.IX/3590 and NIMR/HQ/R.8 C/Vol.I/1973). Written, informed consent was obtained from all participants. In the case of the two participating children aged 11 and 13, formal written consent was obtained from their parent/guardian.

#### Study areas.

Primary data were collected through two participatory workshops held in the two countries of the TIBA partnership (https://tiba-partnership.org). The first workshop took place in Bindura, Zimbabwe (December 2023). Bindura, located in Mashonaland Province in the northeast of Zimbabwe, was selected due to its proximity to NTD-endemic districts, ensuring many affected people could attend the workshop.

The second workshop was held in Zanzibar, Tanzania (March 2024). Zanzibar, an archipelago off the eastern coast of Tanzania, was selected for its accessibility and its known endemicity to lymphatic filariasis and schistosomiasis among other NTDs.

Tanzania has made significant progress towards the elimination of LF and Trachoma, with 89% and 90% of the initially at-risk population, respectively, no longer requiring regular MDA [57]. Although the prevalence of schistosomiasis and STH has declined through school- based MDA conducted for over a decade, persistent transmission remains in some areas [58,59]. Similarly, Zimbabwe has achieved notable reductions in schistosomiasis and STH prevalence through multiple rounds of MDA, though persistent hotspots remain [60]. LF is now reported to be non-endemic in 32 out of the 39 districts previously identified as endemic, indicating a significant decline in endemicity [61]. Trachoma has also markedly improved, with the number of endemic districts dropping from 21 to just 3 in recent years [62]. In both countries, the prevalence of snakebite envenoming is high but remains an underrepresented burden [35,36].

#### Study Participants and Sampling.

Participants were selected using purposive sampling to include two key groups:

i. Affected voices, i.e., people living with the conditions or with lived experiences of the conditions associated with NTDs, and
ii. Various stakeholders involved in implementation of NTD control efforts. This included community health workers, nurses, clinicians, ministry of health policymakers, development partners, researchers from multiple universities, and local media.

Affected individuals were identified through recent programme involvement and community health worker networks, including consultation with the District Medical Officer (DMO), District Environmental Health Officer (DEHO), and District Nursing Officer (DNO). A list of eligible participants was compiled, and individuals were approached at home, provided with study information, and invited to participate. Those who consented were enrolled and interviewed. Stakeholders were invited based on their direct involvement in NTD control and service delivery.

A total of 53 participants took part in the workshop in Zimbabwe, and 37 participated in the workshop in Tanzania. Participant numbers, age ranges and sex distribution by group and country are summarised in Table 1. Affected voices participants had lived with one of four NTDs (trachoma, snakebite envenoming, LF, and schistosomiasis) for approximately 7–20 years and shared first-hand experiences of their chronic manifestations and socio-economic impacts.

**Table 1:** Participant distribution by country, group, age range and sex.

Study Participants	Zimbabwe (N = 53)			Tanzania (N = 37)		
	N	Age range (years)	Sex (M/F)	N	Age range (years)	Sex (M/F)
Affected Voices	24	11-65	9/15	11	22-57	4/7
Health care workers^a^	11	29-57	4/7	9	29-57	2/7
Multi-sector stakeholders^b^	18	21-58	8/10	17	23-58	10/7

^a^Includes community health workers, nurses, and clinicians.

^b^Includes ministry of health policymakers, development partners, researchers from multiple universities, and local media**.**

#### Data collection.

Data were collected through IDIs, FGDs, and stakeholder presentations. All sessions were conducted in English, Shona, and Kiswahili, ensuring inclusive participation and accurate data collection.

#### In-depth interviews (IDIs) and FGDs.

A semi-structured interview guide was developed and pilot tested before data collection commenced. IDIs explored participants’ lived experiences of chronic NTDs, community-level management strategies, and access to health services. Interviews were conducted by teams of two trained facilitators, with one researcher leading the discussion and the second acting as a co-facilitator. The co-facilitator was responsible for taking observational notes, monitoring participant engagement, and providing language interpretation as needed. Interviews lasted between 20 and 30 minutes. FGDs explored shared experiences, perceptions of stigma, barriers to healthcare access, and recommendations for improving NTD-related services. A total of six FGDs, each comprising 8–12 participants, were conducted, with sessions lasting between 60 and 90 minutes.

#### Stakeholder presentations.

During a three-day workshop in Zimbabwe and a two-day event in Zanzibar, all participants delivered short presentations on their lived experiences or professional experiences with chronic NTDs. These sessions were followed by facilitated discussions to explore key issues in more depth. A designated pair of note-takers, one from each country, was tasked with recording main points during presentations and discussions. These notes complemented the audio recordings and were later used to cross-verify themes during the analysis process.

The aspects reported here were part of a larger programme on amplifying the voices of NTD affected people that involved a series of research studies, workshops and public engagement activities [63,64].

For this study we focused on the participant’s experiences of affected people and their health workers in health services in their communities and countries.

#### Data analysis.

All sessions were audio-recorded with participants’ consent, transcribed verbatim, and translated into English where necessary. Data were analysed using thematic analysis following an inductive approach, allowing patterns and themes to emerge from the data. MAXQDA (version 24, VERBI Software, Berlin, Germany) was used to support data organisation, coding, and retrieval. The coding process began with open coding; these initial codes were iteratively refined and grouped into broader categories through constant comparison. Emerging codes were reviewed, and final themes were collaboratively agreed upon by the research team. The thematic analysis focused on identifying recurring patterns across participant narratives. This structured yet flexible approach enabled the research team to manage the complexity of the data while preserving the richness of participant voices and minimising researcher bias [65].

## Results

### Findings from situational analysis

#### Existing policy.

**Fig 2:**
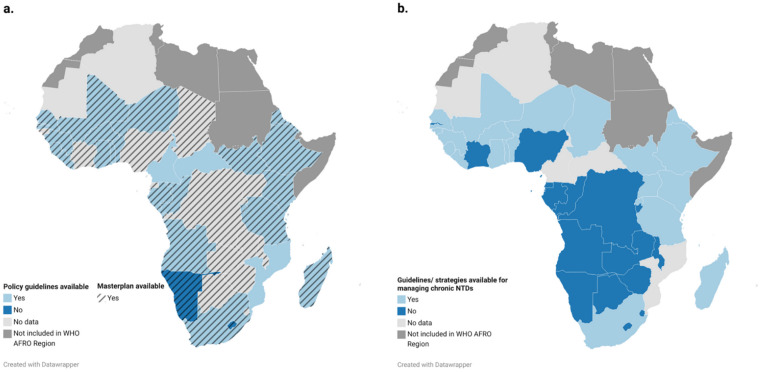
Status of NTD national masterplans and policy guidelines for chronic NTD management across Africa: a) Data from the Global NTD Annual Reporting Form (GNARF; https://www.who.int/en/teams/control-of-neglected-tropical-diseases/data-platforms-and-tools/country-profile); and whether they report having disability management policies. Data visualised using the Datawrapper platform: (base layer of the map: https://www.datawrapper.de/_/KFzxy/) **b)** Data from National NTD Master Plans (https://espen.afro.who.int/tools-resources/documents); countries with NTD masterplans and whether they include policies for managing chronic NTD manifestations. Base layer of the map: https://www.datawrapper.de/_/SGrvz/?v=6. Terms of Use available at: https://www.datawrapper.de/terms.

The situational analysis provided critical insights into the current landscape of health service provision for chronic NTD sufferers across the WHO AFRO region. While countries employ a variety of standard control measures, the integration of care and management for chronic NTD manifestations remains limited. Fig 2 shows the status of national NTD masterplans and policies for managing the chronic manifestations of NTDs across African countries. Using data from both the Global NTD Annual Reporting Form (GNARF) and from national NTD Master Plans, findings showed that only a few countries have strategies in place to address the chronic long-term management of individuals affected by NTDs. Significant gaps persist in policies, healthcare service delivery, and strategically planned long-term management strategies, highlighting the ongoing challenges in ensuring comprehensive and sustained care for chronic NTD sufferers. These findings also highlight inconsistencies in policy frameworks across the continent, emphasising the need for strengthened integration of chronic NTD management into national plans.

#### Standard NTD control approaches.

Most countries rely on a combination of standardised control strategies to manage NTDs, tailored to the specific disease burden in their regions. These include: Preventative chemotherapy (PC), Case Identification and Management, Water, Sanitation, and Hygiene (WASH), Vector Control [66] and Surgery, Antibiotics, Facial cleanliness, and Environmental improvements (SAFE; strategy particularly in trachoma control). While these approaches have been successful in reducing disease prevalence and transmission rates, they largely focus on short-term outcomes and disease elimination, often neglecting the long-term disabilities and chronic conditions that persist after initial treatment.

#### Status of chronic NTD management integration into health systems.

**Fig 3:**
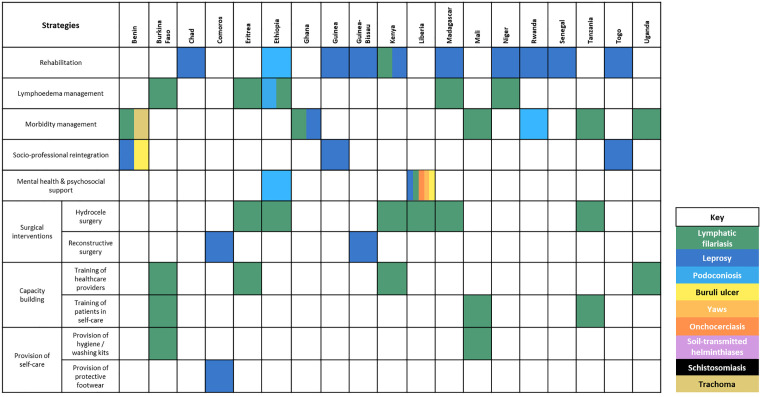
Existing integrated care and management strategies.

The situational analysis highlighted significant variability in the availability of integrated care and management strategies for chronic NTD manifestations across the WHO AFRO region, with only a few countries having strategies in place for integrated care and management [67–85]. As shown in Fig 3, rehabilitation is the most widely implemented strategy, with ten countries providing structured care, particularly for leprosy. However, other critical interventions, such as psychosocial support and socio-professional reintegration, remain largely absent for most NTD-related disabilities, particularly for conditions like lymphatic filariasis, onchocerciasis, [13] and other PC-NTDs. Additionally, mental health and social support interventions which are essential to reduce the social stigma associated with NTDs need to be scaled up [13].

The WHO global report on neglected tropical diseases 2024 [13] highlights that while progress has been made in integrating NTD services into national health plans (28 countries) and providing guidance for managing NTD-related disabilities (19 countries), these efforts remain inconsistent across regions. Implementation of these strategies is inconsistent within countries and remains limited in scale due to financial constraints. While some treatments are provided free of charge, long waiting lists prevent timely access. In most of these countries, integrated care is being implemented but not at full scale, with services available only in select regions or counties, leaving significant gaps in coverage [77,82]. These findings highlight the fragmented and inconsistent approach to chronic NTD care, emphasising the urgent need for broader, standardised, and integrated management strategies across endemic regions.

#### Policy gaps and deficiencies in healthcare services.

Despite global efforts towards integrated healthcare for NTDs, policy implementation remains inconsistent and inadequate. The WHO NTD 2024 report indicates that only 13 out of 49 African countries have established guidelines or policies that address over 75% of the prevalent disabilities associated with NTDs [13]. Notably, only six of the 184 countries considered endemic for at least one NTD had interventions included in national packages of essential services and were budgeted for [13], demonstrating a major gap in policy alignment and integration. While national NTD master plans exist for some countries, their implementation is often limited by weak country ownership, insufficient coordination, inefficient resource use, and slow adoption of new interventions [13].

This policy gap translates into significant unmet needs within healthcare systems, where resources for managing NTD disabilities, such as diagnostics, mental health support, and rehabilitative care, are often lacking [86]. The absence of these essential services exacerbates neglect, preventing individuals with chronic NTDs and related disabilities who require long-term management and support from being fully integrated into mainstream healthcare systems and perpetuating inequalities in access to essential health services.

**Fig 4:**
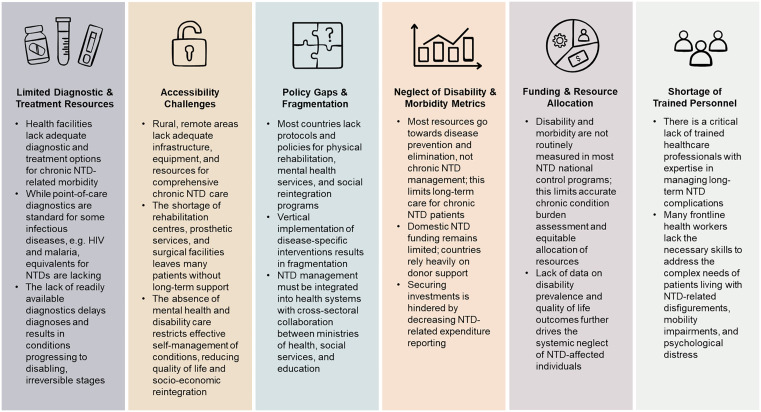
Key gaps from policy guidelines.

In addition to policy gaps, systemic barriers continue to hinder the effective management of chronic NTDs. Key challenges include limited diagnostic resources, fragmented policies, and inadequate access to specialised care, leaving many individuals without essential support. Fig 4 provides a summary of the policy gaps identified from policy guidelines across NTD-endemic countries [67–85,87–108].

#### Social and economic impact.

The economic impact of chronic NTDs remains a major challenge, exacerbating poverty and limiting financial resilience among affected populations. Many individuals affected by NTDs experience catastrophic health expenditures, particularly in resource-limited settings where healthcare services are not covered by insurance schemes. In 2021, an estimated 12.6% of the global population at risk of NTDs encountered catastrophic health spending (i.e., health expenditure exceeding 10%–25% of total household expenditure) allocating a significant portion of their income to treatment costs [13]. Despite efforts to improve financial protection, many endemic regions lack mechanisms at the country level to track household health expenditures, making it difficult to assess the full extent of economic hardship [13]. Therefore, NTD-related disabilities can contribute to long-term unemployment and reduced economic productivity, particularly for individuals with lymphatic filariasis, leprosy, and onchocerciasis, who suffer from mobility restrictions and social stigma [9,86].

### Findings from focus group discussions and in-depth interviews

#### General observations.

The findings from the FGDs and interviews strongly aligned with the situational analysis, further amplifying the identified gaps in chronic NTD care and management. Participants’ first-hand experiences, along with insights from healthcare workers, highlighted persistent challenges, including barriers to healthcare access, policy deficiencies, socioeconomic burden, deep-rooted stigma, and the psychological distress faced by individuals living with these conditions. Their testimonies underscored the urgent need for comprehensive, patient-centred strategies to address these issues.

#### Access to care and treatment.

Participants highlighted critical deficiencies in healthcare service provision, particularly the limited availability of medications, inadequate diagnostic tools, and financial constraints that prevent timely and effective treatment. Particularly, healthcare workers also pointed out the shortage of essential diagnostic tools, limiting their ability to offer effective treatment.

*“Our clinics lack the necessary medications for some of these diseases. We need both medications and comprehensive treatments to effectively help those suffering from these conditions”* (41-year-old female; healthcare worker; FGD, Zimbabwe).

*“Diagnostic tools for diseases like Malaria and HIV are readily available in clinics; the same should be done for LF”* (26-year-old female; LF; IDI, Zimbabwe).

Additionally, participants pointed out structural inefficiencies within the healthcare system, mirroring the policy gaps and healthcare fragmentation highlighted in the situational analysis.

*“Developing a chart for diagnostic criteria and clinical presentations could help clinicians order the right investigations by linking symptoms to likely conditions”* (healthcare worker; Tanzania).

*“The fact that diseases are called neglected diseases, highlights that they should be highly considered”* (40-year-old male: Schistosomiasis; FGD: Tanzania).

Healthcare workers also pointed out that late presentation of cases due to financial difficulties and lack of awareness further complicates effective treatment.

*“Most individuals only go to the hospital/clinic at a later stage when their condition has progressed, often due to lack of funds”* (29-year-old female; healthcare worker; FGD, Zimbabwe).

Healthcare workers recommended community-based interventions to improve early identification and access to treatment.

*“Our NTD control program should be community-based, encompassing the entire population. This approach ensures we catch the little ones early enough to intervene and prevent the progression of pathology from infection”* (47-year-old healthcare worker; FGD; Zimbabwe).

#### Need for a stronger health system support.

Policy weaknesses and lack of funding were consistently raised as barriers to NTD management. Participants called for stronger government action, clearer treatment pathways, and increased financial allocation to ensure sustainable NTD care.

*“Government should increase budget allocation for management of NTDs”* (57-year-old male; District environmental health officer; FGD, Zimbabwe).

Additionally, the need for clearer treatment protocols and referral pathways was emphasised.

*“There is a need to provide clear treatment plans and clear referral pathways so that individuals do not go around in a maze trying to get help”* (Academic/Researcher; FGD, Tanzania).

#### Stigma and social exclusion.

Stigma and discrimination were recurring themes, with affected individuals expressing frustration over being marginalised in their communities. This provided a deeper context into the lived realities of exclusion and discrimination highlighted by the situational analysis.

*“People in the village sometimes laugh at me, but those who understand my condition offer me comfort and support”* (26-year-old female; LF; IDI, Zimbabwe).

*“People often dismiss us in discussions, viewing us as useless because we don’t have children”* (32-year-old female; Schistosomiasis; FGD; Zimbabwe).

Others highlighted how stigma extended to employment and social opportunities, limiting their ability to contribute to society.

*“I have always dreamed of proving my worth to society and achieving something meaningful. However, due to our conditions, we are neglected, and I’ve never truly had the chance to show what I can do or fully enjoy being part of this society”* (22-year-old male; LF; IDI; Zimbabwe).

#### Economic burden and financial hardship.

Participants also highlighted the financial toll of NTDs, including lost income, reduced ability to work, and high out-of-pocket costs. Many participants reported that NTD-related disabilities severely impact their ability to work, leading to financial instability and dependency.

“*I’m not able to carry out most activities that help raise funds for my school-going children. Before the illness, I was farming, but ever since I got sick, I don’t have much energy to do anything, including carrying a bucket of water”* (41-year-old female; Schistosomiasis; IDI; Tanzania).

*“National Health Insurance Fund should be accessible for NTD patients who find it difficult to afford healthcare. I was hospitalised for three months, an experience that was not only expensive but also kept me out of work”* (42-year-old male; LF; IDI, Tanzania).

*“Ever since I was bitten by a snake there is nothing, I am able to do. I am always at home- I wish I could work and be productive like others”* (39-year-old female; snake envenoming; IDI; Zimbabwe).

#### Reproductive health consequences.

The impact of NTDs on reproductive health emerged as a critical but often overlooked issue. Some participants shared their experiences with infertility linked to untreated infections.

*“For years after my marriage, I was not able to conceive. It wasn’t until I participated in a bilharzia research project where they found bilharzia eggs in my uterus. I was treated, and later on, I was able to conceive. Now, I have two children”* (57-year-old female; Schistosomiasis; IDI; Zimbabwe).

## Discussion

This study identifies the persistent gaps in the care and management of chronic NTD-related morbidity and disability in endemic regions. As of May 2025, 56 countries [109] have eliminated at least one NTD. This is tremendous progress in the control of NTDs. However, this progress will not be felt uniformly across the NTD disease profile. This study highlights the inadequacies of healthcare systems in providing long-term support for individuals who have been failed by prevention and treatment and are now consequently living with chronic manifestations of NTDs. Our situational analysis across WHO AFRO countries, all NTD-endemic, revealed deficiencies in policy and policy implementation, fragmented healthcare services, and systemic underfunding, as well as social and economic impact on affected individuals. Our ground-truthing interviews with individuals affected by chronic NTDs in Zimbabwe and Tanzania, along with primary and tertiary healthcare workers and Ministry of Health policymakers, confirmed these gaps in care. The testimonies shared by the participants illustrated not only the persistent barriers to care but also the deep-rooted stigma and psychological distress experienced by those living with these conditions. This systemic neglect perpetuates cycles of poverty, social stigma, and psychological distress, leaving millions of affected individuals without access to comprehensive care and support.

### Health system gaps and policy deficiencies.

A key finding from our study was the inconsistent implementation of NTD policies, particularly regarding chronic morbidity management. While some countries have developed national NTD master plans, their execution remains fragmented and underfunded. The weak integration of morbidity management and disability prevention (MMDP) programs further exacerbates the issue, leaving many individuals without access to rehabilitative care, specialised treatment, or mental health support.

Healthcare workers in our study confirmed that diagnostic limitations, lack of referral pathways, and shortages of essential medicines severely hinder effective NTD management. There is evidence that most NTD-endemic regions lack point-of-care diagnostics and that healthcare professionals are often not equipped to recognise and manage chronic NTD-related disabilities [110,111]. For instance, while diagnostic tools for diseases like HIV and malaria are widely available and integrated into primary healthcare systems, point-of-care diagnostics for chronic NTD conditions such as lymphatic filariasis, schistosomiasis, and snakebite envenoming remain scarce [110]. The absence of standardised treatment guidelines and referral mechanisms further contributes to delays in care, worsening disease outcomes.

A major limitation in addressing chronic NTD-related disabilities is the lack of integration within national health systems [13]. Current morbidity management approaches operate in disease-specific silos, prioritising short-term interventions over long-term patient-centred care. This has the potential to undermine the sustainability of NTD programs. A few countries have set positive examples of sustainability for national NTD programmes. For example, as commitment to achieving universal health coverage, Ghana has developed a comprehensive costing framework to guide the inclusion of NTD services within the national health insurance scheme (NHIS) benefits packages, and revised all NTD management protocols to serve as the basis for the revision of Ghana’s Essential Medicines List and NHIS’ benefits package [42]. To reduce dependence on donor support and boost the sustainability journey towards achieving the WHO road map 2030 targets, Uganda has initiated domestic resource mobilisation, with three districts allocating budgets for NTDs, and plans to increase domestic financing of the total NTD budget from 12% in 2020 to 30% by 2025 [42].

Another critical gap that is key to improving NTD care and management is the lack of education and awareness among both affected individuals and healthcare providers. Significant lack of understanding about NTDs often leads to delays in seeking treatment, and in recognition and diagnosis of NTDs by healthcare workers. As a result, many individuals develop preventable complications that further impair mobility, productivity, and social participation. By providing culturally appropriate health education, this knowledge gap can be bridged. For example, educational campaigns that emphasise the role of adequate WASH practices, as well as preventive medications can significantly reduce diseases such as schistosomiasis and soil-transmitted helminthiasis [112]. Additionally, self-care programs when implemented have been proven to significantly lower costs of care, reduced disability status, and improved quality of life scores [113]. Additionally, community-led peer support networks, as demonstrated with leprosy control and elimination programs [114], should be expanded to other NTDs to foster knowledge-sharing and stigma reduction.

### Social and Economic Consequences of Chronic NTDs.

The socio-economic impact of NTDs remains profound. Chronic NTD-related disabilities disproportionately affect low-income households, trapping affected individuals in cycles of poverty and social exclusion. Participants reported high health expenditures, particularly where healthcare services are not covered by insurance, in line with recent WHO estimates suggests that 12.6% of the global population at risk of NTDs incurs catastrophic health spending [13].

Chronic complications such as mobility impairments, organ damage, and vision loss often lead to permanent disability, limiting individuals’ ability to work, pursue education, or participate in social activities [86]. However, beyond clinical management, rehabilitation services for PC-NTDs are often lacking [115]; the absence of physical therapy, assistive devices, and vocational rehabilitation programs forces many affected individuals into dependence on family members or community support, further deepening economic hardship and social isolation. Integrating disability support programs into existing healthcare structures could significantly enhance functional independence and quality of life [115].

The psychosocial impact of NTD-related disabilities is another critical yet neglected area. Many affected individuals report experiencing stigma, discrimination, and exclusion, particularly those with visible disabilities or mobility impairments [7,51]. Lymphoedema or advanced stages of lymphoedema from lymphatic filariasis, visible skin lesions from leprosy, or blindness from onchocerciasis can lead to ostracization, discrimination, and loss of social status within their communities [116,117]. These experiences are not merely anecdotal; they reflect a broader societal pattern wherein individuals with NTD-related disabilities are marginalised and treated as burdens. Participants in our study recounted instances of being mocked, shunned, and excluded from social and economic activities, leading to profound psychological distress. Yet, mental health services remain largely absent from NTD programs across Africa [51]. Integrating psychological support through counselling, peer-led support groups, and community sensitisation campaigns would help mitigate the psychosocial burden of these diseases and promote greater social inclusion [41,118–120].

### Proposed solutions and way forward

To address the critical gaps identified in this study, we propose a multi-faceted, integrated approach that enhances policy implementation, strengthens healthcare systems, and prioritises community-based interventions.

#### Strengthening policy and health system integration.

National NTD Master Plans should include chronic morbidity management of NTDs. Countries must update their NTD strategies to explicitly incorporate guidelines for managing long-term NTD-related disabilities, including mental health services, rehabilitation programs, and disability-inclusive social protection measures. Governments should also increase domestic health budgets to reduce reliance on external donors and ensure sustainable financing for chronic NTD management. Furthermore, essential NTD services, including treatment for chronic disabilities, should be incorporated into national health insurance schemes to reduce financial barriers to care [14,15].

#### Integrated management of NTDs.

Transitioning from vertical, disease-specific approaches to integrated, person-centred strategies is essential for effectively managing NTDs. Evidence shows that integrating NTD services into existing health systems can improve efficiency and sustainability. For example, incorporating NTD control programs into well-established public health initiatives, such as those targeting HIV/AIDS, malaria, maternal and child health, and WASH, can enhance resource utilisation and service delivery and is more sustainable [121]. The Liberian NTD programme exemplifies this shift by developing an ‘Integrated Case Management Strategy’ that addresses multiple NTDs and their associated morbidities, such as Buruli ulcer, lymphoedema, hydrocele, leprosy, and yaws [122].

#### Enhancing healthcare service delivery.

Building capacity for healthcare workers through expanding NTD-focused training programs can equip health workers with the necessary skills to diagnose and manage chronic conditions more effectively. Alongside this, scaling up affordable, point-of-care diagnostic tools for NTDs will enable earlier detection and treatment, particularly in underserved communities. To ensure comprehensive care, it is also essential that government and non-governmental organisations invest in community-based rehabilitation programs and mental health services to support individuals living with chronic disabilities.

#### Addressing social and economic barriers.

Addressing the social and economic barriers linked to NTDs requires a multi-faceted approach. Public awareness campaigns should focus on reducing stigma and misconceptions surrounding NTDs, particularly through school-based and media-driven interventions. In parallel, economic empowerment initiatives including providing livelihood opportunities, vocational training, and financial support programs for individuals affected by NTD-related disabilities can help mitigate economic hardships. Additionally, integrating reproductive health into NTD programs is critical, particularly through improved screening and treatment for conditions such as Female genital schistosomiasis (FGS), which remain underdiagnosed and neglected within health systems.

#### Call to action from affected voices and stakeholders.

During the FGDs, workshop participants, including affected individuals, health workers, policymakers, ministries of health, and development partners, formulated five key calls to action aimed at improving the management of chronic NTD-related disabilities. These calls emphasise the need for policy recommendations, community advocacy, innovation, systemic integration, and community engagement to ensure inclusive and sustainable interventions for NTDs. Key priorities include developing national guidelines for chronic NTD care, improving healthcare accessibility, enhancing cross-sector collaboration, and strengthening community-driven advocacy efforts. Additionally, the participants highlighted the importance of inclusive decision-making, urging affected individuals to be directly involved in shaping policies that impact their health and well-being. A detailed description of this call to action, along with actionable recommendations to achieve these, will be published separately, providing a comprehensive roadmap to guide stakeholders in implementing these crucial interventions.

## Conclusions

This study highlights the urgent need for a more holistic and integrated approach to NTD management, particularly for chronic disabilities that persist long after initial treatment. Strengthening policy implementation, expanding healthcare infrastructure, and addressing socio-economic barriers are essential steps toward improving the quality of life for individuals living with chronic NTDs. A key priority is ensuring that morbidity management becomes a core component of NTD control programs, breaking the cycle of disability, poverty, and social exclusion.

By adopting patient-centred strategies that incorporate medical, psychological, and social support, NTD-endemic countries can move closer to achieving the WHO NTD Roadmap 2021–2030 [40] and the Sustainable Development Goals (SDGs), particularly Goal 3 (Ensure healthy lives and promote well-being for all at all ages).

Ultimately, by bridging the gaps between policy, healthcare, and community engagement, and prioritising NTD morbidity management, we can ensure that individuals affected by NTDs receive comprehensive, long-term support, and that progress in disease control translates into sustained improvements in well-being.
